# Microbiome–Immune Interaction and Harnessing for Next-Generation Vaccines Against Highly Pathogenic Avian Influenza in Poultry

**DOI:** 10.3390/vaccines13080837

**Published:** 2025-08-06

**Authors:** Yongming Sang, Samuel N. Nahashon, Richard J. Webby

**Affiliations:** 1 Department of Food and Animal Sciences, College of Agriculture, Tennessee State University, 3500 John A Merritt Blvd, Nashville, TN 37209, USA; snahashon@tnstate.edu; 2 World Health Organization Collaborating Centre for Studies on the Ecology of Influenza in Animals and Birds, St. Jude Children’s Research Hospital, Memphis, TN 38105, USA; richard.webby@stjude.org

**Keywords:** highly pathogenic avian influenza, commensal-vaccine vectors, microbiome–immune interaction, artificial intelligence, one health vaccinology

## Abstract

Highly pathogenic avian influenza (HPAI) remains a persistent threat to global poultry production and public health. Current vaccine platforms show limited cross-clade efficacy and often fail to induce mucosal immunity. Recent advances in microbiome research reveal critical roles for gut commensals in modulating vaccine-induced immunity, including enhancement of mucosal IgA production, CD8^+^ T-cell activation, and modulation of systemic immune responses. Engineered commensal bacteria such as *Lactococcus lactis*, *Bacteroides ovatus*, *Bacillus subtilis*, and *Staphylococcus epidermidis* have emerged as promising live vectors for antigen delivery. Postbiotic and synbiotic strategies further enhance protective efficacy through targeted modulation of the gut microbiota. Additionally, artificial intelligence (AI)-driven tools enable predictive modeling of host–microbiome interactions, antigen design optimization, and early detection of viral antigenic drift. These integrative technologies offer a new framework for mucosal, broadly protective, and field-deployable vaccines for HPAI control. However, species-specific microbiome variation, ecological safety concerns, and scalable manufacturing remain critical challenges. This review synthesizes emerging evidence on microbiome–immune crosstalk, commensal vector platforms, and AI-enhanced vaccine development, emphasizing the urgent need for One Health integration to mitigate zoonotic adaptation and pandemic emergence.

## 1. Introduction: The Global HPAI Crisis and Microbiome–Immune Interplay

Highly pathogenic avian influenza (HPAI), particularly H5N1 clade 2.3.4.4b, has triggered unprecedented global epizootics since 2020, resulting in the loss of over 150 million birds in the United States alone and causing widespread economic devastation across the poultry sector [1,2,3,4]. The virus’s rapid antigenic evolution and remarkable geographic spread, highlighted by 297 outbreaks across 26 countries within just April and May 2025, underscore its expanding ecological and epidemiological footprint [4,5,6]. Although traditional vaccines, such as inactivated whole-virus preparations and vectored platforms like a recombinant HPAI-H5 expressed by herpesvirus of Turkeys (HVT-H5) as a vector, have been deployed, they remain suboptimal [3]. Their protection tends to be clade-specific, with cross-clade efficacy dropping below 60% when hemagglutinin sequence similarity declines under 88% [3,7,8]. Moreover, these platforms often fail to induce robust mucosal immunity necessary to block respiratory or fecal–oral transmission and exhibit variable immunogenicity across avian species, for instance, ducks typically display a 3–5-fold lower seroconversion rate than chickens [3,7,8].

Recent discoveries have revealed that the gut microbiome is a key modulator of vaccine-induced immunity in poultry [9,10,11,12,13,14]. Recent studies indicate that specific commensals, such as *Lactobacillus crispatus*, can elicit systemic and mucosal immune responses to suppress virus infections through both TLR-mediated signaling and B-cell activation, while butyrate of a series of active short-chain fatty acid (SCFA) secreted by microbiota species (such as *Faecalibacterium prausnitzii*) significantly reduces viral shedding by various mechanisms including macrophage activation and enhancing CD8^+^ T-cell responses [9,10,11,12,13]. Conversely, microbiota disruption due to antibiotics or heat stress has been associated with impaired mucosal IgA responses and increased mortality following H5N1 exposure [12,13,14]. Additionally, interspecies differences in microbial composition may partially explain immunogenic variability, for example, ducks’ *Proteobacteria*-rich, aquatic-adapted microbiomes exhibit weaker adjuvant effects compared to the *Firmicutes*-dominated communities in chickens, necessitating higher vaccine dosages to achieve comparable immunity [9,10,11,12,13,14].

Beyond poultry, the zoonotic implications of HPAI H5N1 are increasingly concerning. Since 2020, infections have been documented in over 48 mammalian species across 26 countries, with significant mortality events in sea lions and widespread outbreaks among dairy cattle in the United States [15,16,17]. High viral loads detected in bovine milk and conjunctival swabs suggest novel transmission pathways, including occupational exposure among dairy workers [15]. Genetic adaptations such as PB2-E627K (enhancing polymerase activity at human body temperatures), HA-T721A (increasing affinity for human-type receptors), and NA-H274Y (conferring antiviral resistance) are now common in mammalian isolates, elevating pandemic risk [3,15,16,17]. Human infections reported by the WHO show a case fatality rate of 48%, with atypical symptoms including conjunctivitis and severe pneumonia [16,17]. In this context, cross-species microbiome insights—such as the role of segmented filamentous bacteria in viral resistance in mice, or bile acid-modulated immune responses in humans—further emphasize the value of commensal-targeted vaccine strategies [9,10,11,12].

The convergence of the HPAI expanding host range and microbiome-driven immune modulation necessitates a paradigm shift in vaccine design. This review synthesizes five years of advances demonstrating how commensal bacteria act as natural adjuvants (e.g., *Bacteroides*-vectored HA1 delivery), antigen-presenting platforms (e.g., *Lactococcus lactis* displaying H5 trimers), and predictors of vaccine responsiveness (e.g., *Ruminococcus*-associated HI titers) [11,13,14]. Integrating these insights with artificial intelligence (AI)-driven antigen refinement—including machine learning-guided epitope focusing and microbiome-mimicking MAMP (i.e., microbe-associated molecular patterns) fusion—offers a promising path toward broadly protective, mucosa-optimized vaccines [3,13]. However, the escalating spillover to mammals demands urgent “One Health” integration, where avian vaccine strategies must account for interfacial microbiomes—spanning poultry, humans, and wild birds—to mitigate zoonotic adaptation and future pandemic emergence [3,11,13]. With a focus on poultry, this review synthesizes recent years of research on microbiomic regulation of vaccine efficacy and explores emerging strategies leveraging commensal bacteria as vectors, adjuvants, and predictive biomarkers for HPAI control, integrated with AI-driven antigen refinement [15,16,17,18].

## 2. Microbiome–Immune Crosstalk: Implications for HPAI Vaccinology

### 2.1. Cross-Species Microbiome–Immune Lessons

Insights derived from various animal models have highlighted evolutionarily conserved mechanisms of microbiome-mediated immune regulation. These findings provide critical frameworks for improving poultry vaccines against HPAI infections. In murine models, segmented filamentous bacteria (SFB, e.g., ubiquitous commensal *Bacteroides*) have been shown to enhance influenza vaccine responses beyond their known role in rotavirus suppression [18,19,20,21]. They achieve this by engaging RANTES/exotoxin-dependent chemokine cascades, which boost systemic immunoglobulin A (IgA) and facilitate the recruitment of CD8^+^ T-cells to the respiratory mucosa [18,19]. Additionally, the expression of Toll-like receptor 5 (TLR5) in neonatal mice plays a vital role in shaping lifelong microbiota composition. Knockout models have demonstrated that the absence of TLR5 results in impaired antibody responses to influenza vaccination due to a reduction in *Clostridia* populations [18,19,22]. Furthermore, polysaccharide A (PSA) derived from *Bacteroides fragilis* has been implicated in correcting Th1/Th2 imbalances via TLR2 signaling, which reduces post-vaccination inflammation while preserving CD8^+^ cytotoxicity against heterosubtypic influenza strains [18,23,24]. Human studies have provided further insights, revealing that fecal microbiota transplants (FMTs) from high responders to the hepatitis B vaccine can significantly elevate antibody titers in low responders. This effect has been linked to bacteriophage-induced activation of the IL-12/IL-23 axis [25,26]. Additionally, secondary bile acids generated by *Clostridium scindens* have been shown to enhance rabies vaccine responses by promoting T follicular helper (Tfh) cell differentiation through modulation of CXCR5+ dendritic cells [12]. Conversely, exposure to antibiotics, such as azithromycin, has been shown to reduce H1N1 vaccine seroconversion by 48%, which is attributed to the depletion of *Akkermansia muciniphila*, a bacterium that typically enhances TLR-dependent signaling capacity for immune regulation [27,28]. In addition, early-life gut microbiome has been associated with positive vaccine efficacy against infections such as rotavirus, plague and influenza in human infants through various mechanisms (Table 1) [18,19,29,30,31].

Research on wild birds has uncovered mechanisms relevant to HPAI prevention likely analogical to domestic poultry [32,33,34,35,36]. For instance, gut bacteria expressing α-1,3-galactosyltransferase, such as *Escherichia coli* O86:B7, induce natural anti-α-Gal antibodies in *Anas crecca*, providing cross-protection against avian malaria sporozoites via complement-mediated lysis [32,33]. Moreover, shotgun metagenomics of *Grus grus* has revealed *Proteobacteria*-dominated microbiomes carrying a high prevalence of the *mcr-1* gene, indicating environmental antibiotic pressure that may compromise mucosal immunity during migration [34,35]. Stress induced by migration-mimicking conditions has been shown to deplete *Firmicutes* and suppress IL-10 levels in *Branta canadensis*, resulting in increased susceptibility to H5N1 replication in intestinal epithelia [35,36]. Table 1 elaborates on the expanded compendium of microbiome-mediated antiviral mechanisms, summarizing key microbes and metabolites along with their associated immunological outcomes across species [10,18,19,20,21,22,23,24,25,26,27,28,29,30,31,32,33,34,35,36]. For example, *Lactobacillus* species activate TLR-signaling pathways, resulting in a 4.1-fold increase in hemagglutination inhibition (HI) titers post-H5 vaccination in chickens [36]. Other notable mechanisms include the production of Reg3 lectins by segmented filamentous bacteria, which lead to a 90% reduction in rotavirus shedding in mice [10,20,30], and the synthesis of secondary bile acids by *Clostridium scindens*, which promote Tfh and CD8^+^ T-cell differentiation in humans [37,38].

### 2.2. Gut Microbiota as Immunomodulators in Poultry

The avian gastrointestinal tract hosts complex microbial ecosystems, with approximately 10^12^ colony-forming units (CFUs) per gram in chickens and 10^10^–10^11^ CFU per gram in the chicken ceca [11,36]. These microbial communities dynamically shape host immunity through various mechanisms. One key aspect of immune priming involves antibody potentiation, where *Lactobacillus crispatus* upregulates TLR/MyD88-mediated innate immunity and enhances mucosal IgA responses against viral antigens when used as a vaccine delivery platform [11,39]. Concurrently, *Bifidobacterium animalis* relieves inflammatory response and secretes histone deacetylase inhibitors that enhance chromatin accessibility in B-cell loci, thereby boosting antibody affinity maturation. T-cell polarization is another important mechanism, as butyrate produced by *Clostridium Cluster XIVa* induces FoxP3+ regulatory T-cells in the ileal Peyer’s patches [36,40,41]. This action suppresses IL-6-driven inflammation following vaccination and promotes CD8^+^ memory differentiation through the inhibition of HDAC3, sustaining responses to conserved nucleoprotein epitopes. Mucosal barrier fortification is facilitated by IL-22 derived from *Bacteroides uniformis*, which upregulates occludin and claudin-3 expression, thereby limiting HPAI invasion in chicken [11,36]. Commensal metabolites, such as indole-3-acetic acid, are known to downregulate α-2,3-sialic acid receptors in duck enterocytes, consequently limiting viral attachment. Furthermore, metabolites from *Faecalibacterium prausnitzii* regulate cellular inflammation, enhancing IFN-γ production by splenic macrophages in vaccinated broilers and priming rapid IL-18 responses upon heterologous influenza challenge [11,36]. Species-specific variations in microbiota responses have also been observed. Ducks, which have aquatic-adapted microbiomes dominated by *Proteobacteria*, exhibit weaker TLR4 responses compared to chickens, necessitating two-fold higher H5 antigen doses to achieve comparable seroconversion [34,35,36]. In turkeys, the use of butyrate nanogels combined with H7N9 vaccines has been shown to expand antigen-specific T-cells by 5.2-fold through modulation of GPR43-dependent mucosal homing [36,39]. Quail benefit from the combination of *Bacillus subtilis* spores and galactooligosaccharides, which extend heterosubtypic protection for up to 20 weeks by promoting sustained recruitment of IgA+ plasma cells in the jejunal lamina propria [35,42].

Dysbiosis is a significant factor contributing to vaccine failure in poultry. The administration of amoxicillin in Turkish broilers resulted in a 62% reduction in vaccine-induced IgA and a three-log increase in H5N1 shedding due to the depletion of *Faecalibacterium* and *Bifidobacterium*, which are widely recognized as beneficial components of the gut microbiome, playing significant roles in maintaining gut health and influencing the host’s immune system through a variety of mechanisms [11,34,35,36]. Environmental stressors, such as heat stress at temperatures exceeding 35 °C, have been shown to deplete *Firmicutes* by 40%, thereby blunting IFN-γ and granzyme B responses to H5-inactivated vaccines [11,34,35,36]. Concurrent overgrowth of *Escherichia* elevates prostaglandin E2 levels, further suppressing dendritic cell maturation. Additionally, H9N2 infection in chickens has been associated with an 80% reduction in mucin-producing *Lactobacillus* and an expansion of pathogenic *Proteobacteria*, correlating directly with decreased trefoil factor peptides and impaired mucosal healing [11,36]. Table 2 presents an enhanced compendium of microbiota-mediated immune modulation in poultry, detailing the commensal strains, host species, vaccine platforms, immune outcomes, and key mechanisms involved. For instance, *Lactobacillus crispatus* administered in chickens receiving H5N2 inactivated vaccines resulted in a 4.1-fold increase in HI titers through TLR2-dependent B-cell activation, while *Bacteroides uniformis* enhanced mucosal IgA production in turkeys through IL-22 induction in the gut-associated lymphoid tissue (GALT) [11,34,35,36,37,38,39,40,41,42].

### 2.3. Synthesis and Forward Vision

The conserved microbiome–immune mechanisms elucidated across species reveal three translational opportunities for next-generation HPAI vaccines. First, precision probiotics tailored to specific species, such as α-Gal-expressing *E. coli* for ducks or butyrate-producing *Clostridia* for broilers, could enhance cross-clade immunity by mimicking natural immunomodulatory pathways observed in both murine and avian models [12,13,14,15,16,17,36,37,38,39,40,41,42]. In addition, species-specific microbiome variations may affect vaccine design and efficacy, such as chickens harboring *Firmicutes*-dominated communities that produce beneficial short-chain fatty acids, and ducks exhibit more diverse microbiomes with higher *Proteobacteria* abundance [43,44]. These differences affect immune development and vaccine responses, often requiring species-specific vaccination strategies and adjusted antigen doses in waterfowl compared to chickens [45]. Probiotic supplementation with butyrate-producing bacteria can enhance vaccine responses in chickens, while ducks may require modified vaccination protocols [46]. Age-related microbiome maturation further complicates vaccination timing, particularly in young chicks with developing immune systems [47]. Second, AI-guided biomarkers that integrate cecal butyrate levels, *Ruminococcus* abundance, and anti-α-Gal IgM titers could facilitate the prediction of vaccine responsiveness 4–6 weeks prior to vaccination, enabling flock-specific regimen optimization [11,36,45,46,47]. Lastly, engineered commensal vectors, such as CRISPR-edited *Bacteroides ovatus* expressing H5 stem antigens, have demonstrated the potential for increasing survival in H5N6-challenged specific pathogen-free chickens and could be delivered orally through feed, thus overcoming cold-chain limitations in smallholder systems [11,48]. Despite these advancements, critical challenges remain in reconciling species-specific microbial ecology, for example, the *Proteobacteria*-dominant microbiomes of ducks compared to the *Firmicutes*-rich microbiomes of chickens, with universal vaccine platforms [45,46,47]. Future research must prioritize longitudinal studies that map microbiome trajectories during HPAI outbreaks, in conjunction with gnotobiotic models to isolate strain-level immunomodulators. The integration of One Health surveillance, which monitors wild bird resistomes and mammalian spillover sites, will be essential for preempting microbiome-mediated viral evolution [11,36,48].

## 3. Commensal Bacteria as Novel Vaccine Vectors: Emerging Platforms for HPAI Control

Commensal bacteria (including probiotic candidates) offer a transformative approach to vaccine delivery by capitalizing on natural host–microbe interactions for targeted mucosal immunization [49,50]. Their innate mucosal tropism, self-adjuvanticity via microbe-associated molecular patterns (MAMPs), and compatibility with mass delivery methods such as oral or water-based administration make them ideal platforms for poultry vaccination targeting on HPAI prevention [49]. Over the recent years, significant advances have been made in engineering these vectors, optimizing antigen presentation systems, and validating protective efficacy in preclinical and early clinical settings [49,50].

### 3.1. Engineered Probiotic or Commensal Vectors

Among the most developed systems is lactic acid bacteria (LAB), generally including *Streptococcus gordonii, Lactococcus lactis,* or multiple *Lactobacillus species,* which have been successfully used as vaccine vectors to express various virus antigens. The attractive advantages for LAB-based vaccine are known as follows: simple, non-invasive administration (usually oral or intranasal), experimental tractability, its fermentative scalability, and safety profile [51,52]. Particularly, several *Lactobacillus species* have been used to effectively deliver surface-expressed HPAI antigens (e.g., HA or NS1 of H5N1, and HA or M1 and NP of H9N2) to gut-associated lymphoid tissue (GALT), inducing both local (secretory IgA) and systemic (serum IgG) as well as T-cell immune responses in different animal systems including humans, mice, and poultry [51,52,53,54,55,56,57,58]. A recent study in 2023 utilized *Lactococcus lactis*, a safe and food-grade bacterium, to deliver a chicken IgY-Fc-fused HA1 antigen directly to the mucosal tissues, eliciting a strong mucosal and systemic immune response against H9N2 challenges in chickens [59]. Poultry trials confirm the LAB safety and immunogenicity by expression of HPAI antigens, with ongoing work exploring adjuvant effect, cytokine or antigen co-expression, and lyophilization for feed-based delivery [51]. Despite these promising results, the platform remains in preclinical development due to concerns over biocontainment and the technical challenges of anaerobic cultivation. Kill-switch systems and scalable feed-compatible formulations are under investigation [49,50].

Spore-forming *Bacillus subtilis* offers practical advantages for vaccine formulation and stability. Spores survive feed pelleting and deliver secreted antigens to the ileum and ceca, evading degradation by gastric acid [60]. A study involving the in-ovo administration of a *Bacillus subtilis* strain engineered to express hemagglutinin 1 (HA1) and neuraminidase (NA) fusion proteins in broilers demonstrated promising results in protecting against a highly pathogenic avian influenza (H5N8) challenge [61,62]. Specifically, the study observed a 70% survival rate among the broilers that received this treatment when later challenged with the H5N8 virus [62]. The enhanced survival rate in this study was attributed to the robust immune responses generated by the *B. subtilis* strain expressing the HA1-NA fusion proteins [61,62]. The researchers observed increased levels of ileal IgA and IFN-γ, indicating improved mucosal and cellular immunity, respectively [61,63]. This suggests that the administration of the recombinant *B. subtilis* stimulated local immune responses in the intestinal tract, a crucial barrier against pathogens like avian influenza viruses [61,62]. Previous research has demonstrated the potential of recombinant *B. subtilis* as a delivery system for vaccine antigens, inducing both mucosal and systemic immunity in chickens. While promising, it is important to note that the studies concluded that the recombinant *B. subtilis* did not achieve the same level of protection as commercial vaccines. However, the findings suggest that this approach has significant potential as a biological agent for protecting chickens from avian influenza, and further research may lead to enhanced efficacy. This vector is commercially available as an adjuvant or probiotics (e.g., Alterion^®^), and next-generation strains are being engineered to co-express antigens and beneficial metabolites like butyrate [64].

**Figure 1:**
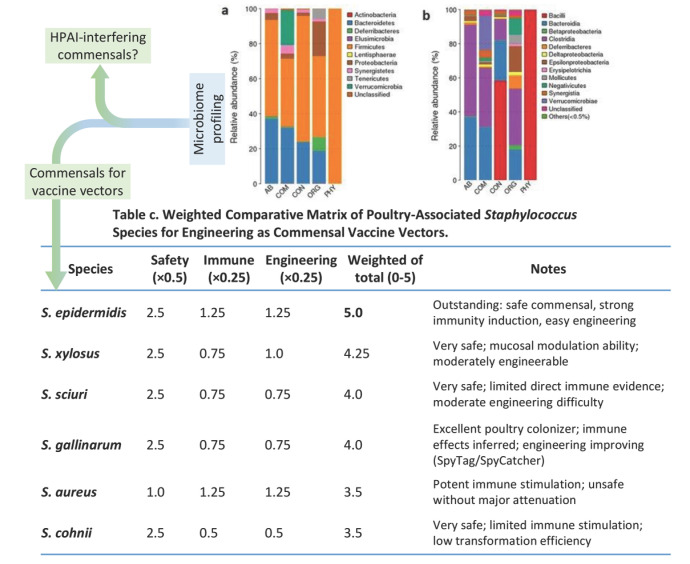
**Targeted microbiome profiling for selecting commensal bacteria with HPAI-interference activity and vaccine vector potential.**  ***Upper panel***: (**a**,**b**) Certain gut commensal bacteria have been shown to enhance antiviral resilience through mechanisms such as maintaining epithelial homeostasis, expelling pathogens, and stimulating innate immune responses, particularly interferon (IFN) production [18,19,26]. However, effective poultry commensal species/strains capable of interfering with highly pathogenic avian influenza (HPAI) remain largely understudied. We propose to integrate meta-analysis and metagenomic profiling approaches to identify 2–4 poultry commensal strains with demonstrated HPAI-interference potential. ***Bottom panel**:* In particular, select skin-derived *Staphylococcus* species/strains will be engineered as vaccine vectors to surface-display HPAI antigens efficiently. Preliminary validation (Panel **c**) shows our AI-optimized workflow for prioritizing *Staphylococcus* candidates based on weighted scores emphasizing commensal safety (50%), immune stimulation capacity (25%), and genetic engineering tractability (25%), yielding a weighted total score out of 5. See references for details on the meta-analysis, species selection, and predictive scoring pipeline [49,50,51,52,53,54,55,56,57,58,59,60,61,62,63,64,65,66,67].

Another frontier involves skin commensals such as *Staphylococcus epidermidis*, which engage cutaneous immunity by activating Langerhans cells via TLR2 signaling. When engineered to display extraneous antigens fusing to the bacterial Aap domain, this bacterium induces neutralizing IgG against expressed antigens and exerts a vaccine effect in mice, with protection linked to dendritic cell activation in draining lymph nodes [65,66]. Therefore, *Staphylococcus*-based vectors may work through the skin or mucosal pathways to intrinsically enhance immune responses by activating TLR2 pathways and stimulating mucosal IgA, Th1/Th17 polarization, and interferon-stimulated genes (ISGs), which are crucial for antiviral defense for vaccine-design against HPAI in poultry. We recently performed meta-analyses to profile poultry microbiomic datasets and weighted poultry-associated *Staphylococcus* species for engineering as commensal vaccine vectors using an AI-facilitated comparative matrix (Figure 1). This matrix prioritizes host safety (weighted at 50%), while immune stimulation (Immune) and genetic engineering tractability (Engineering) are each weighted at 25% to reflect their importance for vaccine efficacy and feasibility. The analysis pinpoints that several poultry commensal *Staphylococcus* species are promising commensal vectors for leveraging as microbiome-based interventions against evolving avian viruses (Figure 1). While still in early preclinical stages, efforts are underway to adapt the commensal vector system for poultry via skin colonization and/or nasal spray delivery formats [65,66].

### 3.2. Microbiome-Targeted Adjuvants: Synbiotics and Postbiotics

Beyond live bacterial vectors, microbiome-derived products, such as postbiotics and synbiotics, offer complementary immunomodulatory effects that enhance vaccine efficacy. A recent study indicates that lactic acid bacteria produce lactate, which can play an adjuvant role in modulating trained immunity through metabolic and epigenetic rewiring in immune monocytic cells [68]. Postbiotic formulations like butyrate nanogels derived from *Clostridium Cluster XIVa* improve CD8^+^ T-cell cytotoxicity via HDAC inhibition and may enhance mucosal immunity when co-administered with HPAI vaccines [69,70]. Bacteria of microbiota (e.g., *Vibrio cholera* and *Bdellovibrio bacteriovorus*) possess enzymes similar to the mammalian cyclic GMP-AMP synthase (cGAS), which can synthesize cyclic dinucleotides (CDNs), including cyclic GMP-AMP (cGAMP) to protect bacteria against viral infection [71]. A recent study has shown that cGAMP, as an agonist of cGAS-STING pathway, when used as a mucosal adjuvant with an inactivated H7N9 vaccine, significantly enhanced humoral, cellular, and mucosal immune responses, providing complete protection against homologous virus challenge and inducing cross-protective immunity against H1N1, H3N2, and H9N2, highlighting its potential as a broad-spectrum adjuvant for pre-pandemic influenza vaccines [72]. Similarly, polysaccharide from *Atractylodes macrocephala* significantly enhanced the HI titer, IgG and specific sIgA levels to reduce viral loads in H9N2-vaccinated chickens [73].

Synbiotic strategies that combine probiotics with targeted prebiotics further enhance protective immunity. Combinations of *Bacillus subtilis* spores and galactooligosaccharides (GOSs) promote cecal butyrate production and support heterosubtypic protection for up to 20 weeks in broiler chickens [74]. The enhancement correlates strongly with elevated cecal butyrate levels. Another promising approach involves *Lactobacillus crispatus* paired with mannan-oligosaccharides (MOSs), which activates TLR-MyD88 signaling and alters epigenetic modification in chicken cecal tonsils to potentiate poultry immunity when combined with HPAI-targeted vaccination [68,75,76].

### 3.3. Challenges and Translational Considerations

Despite promising preclinical results, critical barriers impede the translation of microbiota-based HPAI vaccines from laboratory to commercial application. These barriers include the following: (1) Biocontainment and safety: Genetically engineered bacterial vectors pose significant environmental risks. Containment failures with genetically modified organisms in research settings have highlighted the need for robust biosafety measures [77]. Current mitigation strategies include using non-standard amino acid, though these require extensive validation to prevent evolutionary escape [77,78]. The European Food Safety Authority (EFSA) mandates comprehensive risk assessments for genetically modified bacterial vectors, including physical barriers, genetic safeguards, and environmental monitoring protocols [79,80]. (2) Species-specific efficacy challenges: Real-world deployment reveals critical species variations in vaccine responses. Fundamental differences exist between avian species’ gut microbiomes—chickens typically harbor *Firmicutes*-dominated communities while waterfowl exhibits more diverse microbiomes with higher *Bacteroidetes* abundance [44,81,82]. These microbiome differences significantly influence immune responses and vaccine efficacy across species [43]. Consequently, researchers are developing species-specific or broadly protective approaches, though this multiplies production costs and regulatory complexity [83,84,85]. (3) Manufacturing and scalability bottlenecks: Industrial scaling presents substantial technical hurdles. Bacterial spore vaccines show promise but face challenges in large-scale production due to complex fermentation requirements and quality control issues [86]. Live bacterial vaccine systems require cold-chain maintenance throughout production and distribution, significantly increasing costs compared to conventional inactivated vaccines [87]. Production of specialized bacterial vectors remains expensive, with cost-effectiveness being a major barrier to commercial adoption [88]. (4) Regulatory and implementation barriers: Novel bacterial vectors face extensive regulatory scrutiny due to concerns about horizontal gene transfer and environmental release [89]. Field implementation in resource-limited settings reveals additional challenges including cold-chain requirements, farmer acceptance, and infrastructure limitations that affect vaccine efficacy [90]. Regulatory fragmentation across countries creates significant market approval delays, particularly problematic for emergency outbreak responses [79,80,91]. Success requires coordinated solutions including thermostable formulations, harmonized regulatory frameworks, and cost-reduction through platform technologies enabling rapid adaptation for emerging strains [90,91,92].

## 4. AI-Driven Microbiome Engineering for Vaccine Refinement and Viral Surveillance

Artificial intelligence is revolutionizing microbiome-based vaccine development through validated computational tools that enhance antigen design, predict microbial interactions, and enable real-time monitoring of viral evolution. This rapidly evolving field shows promise for various biomedical applications, and several validated models relevant to microbiome studies and vaccine development are briefed here.

*Validated AI Models for Antigen Design:* EVEscape, a deep learning model developed by Bloom et al., has demonstrated accuracy in predicting viral escape mutations, with validated performance on influenza and other RNA viruses [93]. This model shows promise for forecasting antigenically significant mutations in viral surface proteins, though its application to microbiome-based vaccine platforms remains in early development stages. AlphaFold and related protein structure prediction tools have shown utility in epitope identification and vaccine design [94,95,96]. Machine learning approaches for epitope prediction have demonstrated improved accuracy over traditional methods, particularly for conserved regions suitable for cross-protective vaccines [97]. These AI-guided approaches can significantly reduce experimental screening time while improving antigen stability in bacterial expression systems [98,99].

*Machine Learning for Microbiome Optimization:* Convolutional neural networks and other machine learning approaches are increasingly applied to microbiome analysis, showing promise for predicting bacterial colonization patterns and host–microbe interactions [100]. These models can identify optimal probiotic strains for specific applications, though species-specific validation remains essential [101]. Random forest and other ensemble algorithms have shown utility in analyzing metabolomic and microbiome data to predict immune responses, enabling more personalized vaccination strategies [102]. Such approaches could potentially enable real-time dose adjustments based on individual host characteristics [103].

*Real-Time Viral Surveillance Integration:* GISAID and other genomic surveillance platforms increasingly incorporate automated analysis tools for rapid variant detection and characterization [104]. Machine learning approaches can accelerate phylogenetic analysis and variant identification compared to traditional methods [105]. Time-series modeling and environmental surveillance data show promise for outbreak prediction, though validation across diverse settings remains limited [106]. Integration with vaccination strategies could enable more targeted and preemptive responses to emerging threats [107,108,109].

Current AI applications face significant validation challenges, particularly when applied to novel situations lacking training data [93]. Model performance often decreases when applied to new viral clades or host species, requiring continuous retraining and validation [108]. Cross-validation studies indicate that ensemble approaches combining multiple models often improve robustness compared to individual algorithms [109]. However, continued validation across diverse field conditions remains essential for widespread adoption of AI-driven vaccine development tools [108]. The integration of these AI tools represents progress toward precision vaccinology, though significant challenges remain in translating computational predictions to effective field applications.

## 5. Future Perspectives and One Health Integration

The convergence of the HPAI expanding zoonotic potential and microbiome-driven vaccine technologies necessitates integrated One Health strategies that coordinate microbiome-based interventions across wildlife, livestock, and human health sectors to prevent pandemic emergence.

First, it might coordinate microbiome-based surveillance and intervention. One Health integration of microbiome-based HPAI vaccines requires coordinated surveillance systems that monitor viral evolution alongside host–microbiome dynamics across species boundaries. Wild bird populations serve as natural reservoirs for influenza viruses, with their gut microbiomes influencing viral persistence and transmission potential [110,111]. Understanding these microbiome–virus interactions across migratory flyways can inform strain selection for poultry vaccines and predict zoonotic adaptation risks [112,113]. The East Asian–Australasian Flyway Partnership provides a framework for such integrated surveillance, though incorporation of microbiome data and coordinated vaccine deployment remains limited [113]. Future systems could integrate real-time monitoring of both viral genetics and host–microbiome composition to predict outbreak risks and guide preemptive vaccination strategies using microbiome-based platforms.

Second, it facilitates cross-species microbiome platform development. Engineered commensal vectors like *Lactococcus lactis* and *Bacillus subtilis* offer potential for cross-species vaccine platforms that could protect both livestock and wildlife populations at critical interfaces [15,16,17]. However, species-specific microbiome variations require careful adaptation of these platforms, for example, optimizing *Bacteroides*-based vectors for waterfowl while maintaining *Lactococcus* systems for terrestrial poultry [43,44]. In this regard, AI-driven tools can accelerate this cross-species platform development by predicting optimal bacterial characteristics for probiotics and vaccine vector suitably for different host microbiomes [93,108,109]. Such predictive modeling could enable rapid deployment of species-appropriate vaccines during cross-species transmission events, potentially interrupting zoonotic adaptation before human pandemic emergence.

Third, microbiome interventions may mitigate epidemic and zoonotic risks. Critical human–animal interfaces, particularly in poultry processing facilities and live bird markets, represent high-risk zones for zoonotic transmission where coordinated microbiome interventions could provide protection [114]. Probiotic strategies that enhance mucosal immunity in both animals and exposed human populations could create “immune barriers” against emerging epidemic and zoonotic adaptation [115]. The concept of shared microbiome-based immunity across species boundaries remains largely theoretical but represents an important direction for pandemic prevention research. Such approaches would require careful safety evaluation and regulatory coordination across veterinary and human health authorities [116,117,118].

Fourth, it applies to the regulatory and implementation framework of One-Health. Successful One Health integration of microbiome-based HPAI vaccines requires harmonized regulatory frameworks that can rapidly approve and deploy these interventions during emerging outbreak situations. The WHO–FAO–WOAH tripartite collaboration provides a foundation, though specific protocols for microbiome-based interventions need development [119]. Field deployment challenges identified in previous sections—including cold-chain requirements, species-specific efficacy variations, and manufacturing scalability—become more complex in One Health contexts requiring coordination across multiple species and jurisdictions [120,121].

Finally, AI application enhances pandemic preparedness. AI-driven surveillance systems that integrate viral genomics, host–microbiome data, and ecological factors could provide early warning of pandemic-prone viral variants [122]. These systems could automatically trigger deployment of appropriate microbiome-based vaccines across relevant species populations, potentially interrupting pandemic emergence at its source. Machine learning models trained on cross-species transmission data could predict optimal intervention strategies, including which microbiome-based platforms to deploy at specific wildlife–livestock–human interfaces [93,97]. Therefore, future research must address the following: (1) safety and efficacy of microbiome-based vaccines across species boundaries, (2) development of thermostable formulations suitable for diverse deployment conditions, (3) regulatory pathways for rapid emergency deployment, and (4) integration of microbiome interventions with existing surveillance and control systems. This will benefit the ultimate goal to create resilient, ecosystem-level immunity that prevents HPAI variants from achieving pandemic potential through coordinated microbiome-based interventions guided by AI-enhanced surveillance and deployed through integrated One Health systems. While many of these approaches remain largely theoretical or preclinical, they represent important directions for future research and development in pandemic preparedness and response strategies.

## Figures and Tables

**Table 1:** Examples of cross-species microbiome-mediated antiviral mechanisms [10,18,19,20,21,22,23,24,25,26,27,28,29,30,31,32,33,34,35,36,37,38].

Mechanism	Key Microbes/Metabolites	Immunological Outcome	Species
Increase anti-HA1-specific IgA and IgG levels	*Lactobacillus* spp.	↑ HI titers post-HA1 vaccination	Mouse/ Chicken
Reg3γ lectin production	Segmented filamentous bacteria	↑ IFN and Th17 response, ↓ rotavirus shedding	Mouse/ Pigs
Secondary bile acid synthesis	*Clostridium scindens*	↑ Tfh cell differentiation	Human
SCFA such as Butyrate induction	*Faecalibacterium*	↑ CD8^+^ cytotoxicity vs. H5N1	Duck/ Chicken
α-Gal epitope presentation	*Escherichia coli* O86:B7	Complement-mediated sporozoite neutralization	Eurasian teal
PSA-TLR2 signaling	*Bacteroides fragilis*	Correction of Th1/Th2 imbalance	Mouse
Virome-IL-12 axis	*Caudovirales* bacteriophages	Enhanced plasmablast differentiation	Human

**Table 2:** Microbiota-mediated immune modulation of poultry vaccines [11,36,37,38,39,40,41,42].

Commensal Strain	Host Species	Vaccine Platform	Immune Outcome	Key Mechanism
*Lactobacillus crispatus*	Chicken	H5N2 inactivated	↑ HI titers	TLR2-dependent B-cell activation
*Bacteroides uniformis*	Turkey	rHVT-H5 vectored	↑ mucosal IgA	IL-22 induction in GALT
*Faecalibacterium prausnitzii*	Chicken	DNA vaccine (H5)	↓ viral load	Butyrate-enhanced CD8^+^ cytotoxicity
*Bifidobacterium* *animalis*	Duck	H5 mRNA-LNP	↑ neutralizing IgG	HDAC inhibition in germinal centers
*Clostridium Cluster XIVa*	Quail	H7 VLP	↑ Treg/Th17 balance	SCFA-GPR43 signaling
*Bacillus subtilis* + GOS	Chicken	rNDV-H5 vectored	↑ heterosubtypic protection	Galectin-9-mediated T-cell memory
*Akkermansia* *muciniphila*	Duck	Oral vector vaccine	↑ antigen uptake	Mucin layer modulation
